# Community Pharmacy Staff’s Knowledge, Educational Needs, and Barriers Related to Counseling Cancer Patients and Cancer Survivors in Denmark

**DOI:** 10.3390/ijerph20032287

**Published:** 2023-01-27

**Authors:** Caroline Buhl, Nadia Lund Olsen, Lotte Stig Nørgaard, Linda Aagaard Thomsen, Ramune Jacobsen

**Affiliations:** 1 Department of Pharmacy, University of Copenhagen, 2100 Copenhagen, Denmark; 2 Science to Society, Danish Cancer Society Research Center, 2100 Copenhagen, Denmark

**Keywords:** cancer, educational needs, barriers, community pharmacy, pharmacists, pharmaconomists, Denmark

## Abstract

Objective: The study aimed to determine Danish community pharmacy staff’s knowledge, educational needs, and barriers when communicating with cancer patients/survivors. Furthermore, the study investigated whether pharmacy staff was interested in participating in education about cancer. Methods: A cross-sectional questionnaire survey was conducted among community pharmacy staff (pharmacists and pharmaconomists) in Denmark. Descriptive and bivariate (t-test and chi-square) statistics were used to analyze the data. Results: In total, 134 staff members responded to the questionnaire. Their self-reported knowledge of cancer-related topics was between ‘very little knowledge’ and ‘some knowledge’. The most well-known topics concerned risk factors for cancer and side effects from cancer treatments. The importance of learning more about the same topics was rated between ‘important’ and ‘very important’. The largest barriers identified in counseling cancer patients/survivors were a lack of knowledge about cancer, a focus on healthcare problems other than cancer, and a traditional view of community pharmacies as a place to pick up medication. Pharmacy staff expressed interest in participating in educational programs about cancer treatment (91.0%), communication with cancer patients (88.1%), and late effects of cancer (93.3%). Conclusion: Community pharmacy staff show interest in participating in education regarding cancer, but need more knowledge to properly counsel cancer patients and survivors at the community pharmacies. This important barrier should be addressed in future educational programs for community pharmacy staff.

## 1. Introduction

Cancer is a leading cause of death; in 2020, it accounted for nearly 10 million deaths worldwide [1]. In Denmark, one in three persons will be diagnosed with cancer before the age of 75, and as of 2020, 362,715 Danes were living with or had survived cancer. This number is expected to increase because of the increasing prevalence of cancer and the increasing survival following better treatment options [2].

The Pharmaceutical Group of the European Union (PGEU) have suggested several recommendations for meeting the needs of Europe’s patients and healthcare systems in their fight against cancer. One of them is to increase access to healthcare services close to where people work or live. This could be assured by involving community pharmacy staff in providing support to cancer patients throughout their entire cancer trajectory [3]. Community pharmacists are recognized by the World Health Organization (WHO) as some of the most easily accessible healthcare professionals for patients to get in contact with without an appointment [4].

In Denmark, both pharmacists and pharmaconomists (the Danish counterpart to pharmacy technicians in other countries) are employed at community pharmacies. The pharmacist program is a 5-year (300 ECTS) MSc in Pharmacy and the pharmaconomist program is a 3-year (180 ECTS) vocational training program [5,6]. Pharmacists are specialists in medicines and minor illnesses [7] and are taught the most about medications compared to other healthcare personnel in Denmark. Pharmacists are not taught as much as pharmaconomists, though, on how to communicate knowledge about medication to patients [5]. The role of pharmaconomists is extensive and bears some resemblance to those of pharmacists in other countries [8,9].

The growing role of community pharmacies is clearly defined by the different healthcare services they provide around the world. In Denmark, community pharmacies offer several healthcare services that are standardized and available from all pharmacies; they resemble services available in Western Europe and North America. These services support medication safety and prevent disease by supporting the rational use of medicines [10]. Community pharmacy-based services with a focus on patient-centered care have been shown to improve health and the quality of life, lower mortality rates, and be cost-saving [4]. It is also well documented that counseling and healthcare services offered by Danish pharmacies contribute to improved patient self-care, physical health status, treatment adherence, medicine knowledge, and satisfaction with medical treatment [11,12].

International experience shows that community pharmacies can be successfully included in healthcare services for cancer prevention and cancer care: examples being, smoking cessation, nutrition advice, health promotion, recommending and administering vaccines [3,13,14], cancer detection through screening [13,14,15], as well as dispensing oral chemotherapy [16,17]. However, there are currently no standardized healthcare services specifically targeting cancer patients and/or survivors offered at Danish community pharmacies.

### Objective of the Study

Contributing to the development of a healthcare service for cancer survivors in Danish community pharmacies, this study aimed at describing Danish pharmacy staff’s knowledge and educational needs regarding cancer treatment modalities and organization of cancer care and barriers experienced by pharmacy staff when communicating with cancer patients or survivors in the community pharmacy. Furthermore, the study investigated whether pharmacy staff was interested in participating in education about cancer.

## 2. Materials and Methods

### 2.1. Questionnaire Development

A cross-sectional survey was conducted. To develop the questionnaire, the literature was searched for validated questions from published studies reflecting the aims of the current study. The identified questions were adjusted and specified to a Danish context by the research group which consists of the authors of this paper. The final questionnaire consisted of four parts: part one concerned pharmacy staff’s self-perceived knowledge and educational needs; part two concerned barriers when counseling cancer patients at the community pharmacy; part three concerned the respondents’ interests in participating in education regarding cancer; and part four concerned demographics of the respondents.

Part one on pharmacy staff’s self-perceived knowledge and educational needs consisted of sections devoted to the cancer trajectory and cancer treatment, the organization of cancer care, and the most common cancer types. The majority of the questions were inspired by an Australian questionnaire by Hussainy et al., concerning palliative care for cancer patients, and edited so that the questions related to cancer in general [18]. Questions concerning late effects of cancer, the national cancer plan, and the most frequently occurring cancer types in Denmark, were added [2,19,20]. The respondents rated their level of knowledge on a 5-point Likert scale from 1 = No knowledge to 5 = In-depth knowledge, and the importance of learning more about the topics if an educational program was to be constructed on a 5-point Likert scale from 1 = Not important to 5 = Extremely important. The questions concerning knowledge were organized chronologically in the order of cancer disease progression.

In part two, the questions concerning barriers were generated based on six different studies describing barriers for community pharmacies [13,21,22,23,24,25], related to breast cancer [21,23], dispensing anti-cancer drugs [22], screening cancer patients [13,24], and communicating with cancer patients [25]. The questions concerning barriers were edited to present statements about barriers when managing cancer in general and accordingly grouped into two sections on barriers related to the pharmacy environment and organization and barriers related to pharmacy staff’s knowledge and attitudes towards cancer, cancer treatment, and communication with cancer patients. The respondents were asked to rate how much they agreed with each of the listed statements on a 5-point Likert scale from 1 = Strongly disagree to 5 = Strongly agree. The statements on barriers were presented in a random order.

In part three, questions concerning participants’ interest in participating in education regarding three cancer-related topics were rated on a 5-point Likert scale from 1 = Not interested at all to 5 = Very interested.

In part four, questions concerning demographics were mainly close-ended.

The questionnaire was discussed and adjusted in two rounds by the research group. Afterwards, the questionnaire was piloted with a psychology student, two pharmacy students, and two community pharmacists in three rounds of cognitive interviewing using the ‘think-aloud’ method [26,27,28], as suggested by Tourangeau [26,29] (see Table 1). The results from the cognitive interviews were analyzed and the questionnaire was modified by specifying the wording of questions and items brought up in the pilot study. Using the idea of category saturation, the number of rounds was three [28].

The final survey consisted of 84 questions, with 10 close-ended questions, three open-ended questions, and 71 Likert scale items. The full survey could be completed within 15 min and can be found in the Appendix A. Internal consistency reliability, Cronbach’s alpha for knowledge, educational needs, and barriers items were respectively 0.96, 0.93, and 0.79.

### 2.2. Sampling

The data collection was conducted using SurveyXact, an online survey system, via emails, including a link to the questionnaire, and addressed to all 452 pharmacies and pharmacy branches in Denmark. The questionnaire was also posted on two Facebook groups for pharmacy staff in Denmark. To ensure that only community pharmacy staff (pharmacists, pharmaconomists, and students of either profession [6]) answered the questionnaire, a filtering question was added to the beginning of the questionnaire (“Are you currently working at a community pharmacy in Denmark?”). The data were collected between 2 November and 30 November 2021.

### 2.3. Analysis

Only complete data were analyzed. The Likert scale responses were given a number from 1–5, and the mean and standard deviation (with a 95% confidence interval (CI)) was determined. A comparison of pharmacists’ and pharmaconomists’ responses was made by applying an independent samples *t*-test. When comparing categories of multiple-choice responses, a Pearson Chi-square test of independence (χ^2^) was used. The data were analyzed using IBM Statistical Package of Social Sciences (SPSS) version 28. The figures were depicted using Microsoft Excel.

### 2.4. Ethical Considerations

The study did not collect data on the respondents’ health [30,31]. All respondents were informed in writing about the aims of the study and their right to withdraw from the study and provided informed consent before participating in the study [32,33]. Data was stored according to the policy of the University of Copenhagen which follows the national legislation and the requirements of the General Data Protection Regulation (GDPR) [30]. According to Danish law, a formal ethical assessment was not necessary, as the study did not collect any biological material.

## 3. Results

### 3.1. Characteristics of Respondents

In total, 175 pharmacy staff members responded to at least one question in the questionnaire. After excluding respondents with missing data, the final study population consisted of 134 pharmacy staff members, which corresponds to approximately 5% of all pharmacy staff in Denmark [34]. Characteristics of the respondents are summarized in Table 2. The majority of the respondents were female (83.6%), younger than 45 years (65.7%), and pharmacists (59.0%). The respondents possessed different job titles in a pharmacy and the pharmacies were located in all five Danish regions. The respondents had less than 1 year to more than 20 years of experience working in a Danish community pharmacy.

The respondents were asked about the origin of their cancer knowledge. The results are shown in Table 3. Most of the pharmacists had obtained their knowledge of cancer from their pharmacy education (63.3%) and most of the pharmaconomists had obtained their knowledge from personal experience or cancer experienced by close relations (64.7%).

### 3.2. Knowledge and Education Needs

Table 4 shows the mean ratings of the respondent’s level of knowledge and the importance of learning more about 13 topics related to the cancer trajectory and cancer treatment and eight topics related to the organization of cancer care.

The respondents’ self-perceived knowledge was on average rated higher for the topics related to the cancer trajectory and cancer treatment than for the topics related to how cancer care and rehabilitation are organized in Denmark. The respondents’ self-perceived level of overall knowledge was valued between ‘very little knowledge’ to ‘some knowledge’. Only for ‘causes and risk factors for getting cancer’ (mean = 3.4); ‘side effects of medical treatment of cancer’ (mean = 3.3), and ‘screening for cancer’ (mean = 3.0) the respondent’s mean self-perceived knowledge scores were above 3.0 (scale range 1 to 5), which corresponded to the ratings between ‘some knowledge’ and ‘a lot of knowledge’.

When rating the importance of learning more about cancer, all topics except ‘incidence and prevalence of cancer’ (mean = 2.6), ‘surgical treatment of cancer’ (mean = 2.7), ‘radiation therapy for cancer’ (mean = 2.9), and ‘diagnosis of cancer’ (mean = 2.9) received an average score above 3.0 meaning that they were regarded as important/very important/extremely important. ‘Cancer screening’, ‘surgical treatment of cancer’, ‘drug interactions during cancer treatment’, ‘management of late effects after cancer and/or cancer treatment’, and ‘supplementary use of herbal remedies or supplements’ were topics with a statistically significant discrepancy in ratings for existing knowledge and importance for gaining more knowledge assessed based on confidence intervals (see Table 4).

### 3.3. Differences between Pharmacists and Pharmaconomists

The pharmacists rated their knowledge of the cancer trajectory and cancer treatment significantly higher than the pharmaconomists did for the topics ‘causes and risk factors for getting cancer’ (*p* = 0.001), ‘complications after surgery and radiation therapy’ (*p* = 0.008), stages of cancer (*p* = 0.010), ‘diagnosing of cancer’ (*p* = 0.004), ‘side effects of medical treatment of cancer’ (*p* = 0.046), ‘medical treatment of cancer’ (*p* = 0.050), and ‘incidence and prevalence of cancer’ (*p* = 0.047). The pharmacists rated their knowledge of how cancer care and rehabilitation is organized in Denmark significantly higher than the pharmaconomists for the topics ‘treatment of cancer’ (*p* = 0.009), ‘diagnosis of cancer’ (*p* = 0.001), and ‘overall knowledge about the national cancer plan’ (*p* = 0.010). No significant difference was found between the pharmacists and the pharmaconomists regarding topics they would like to learn more about (see Table 4).

**Figure A1:**
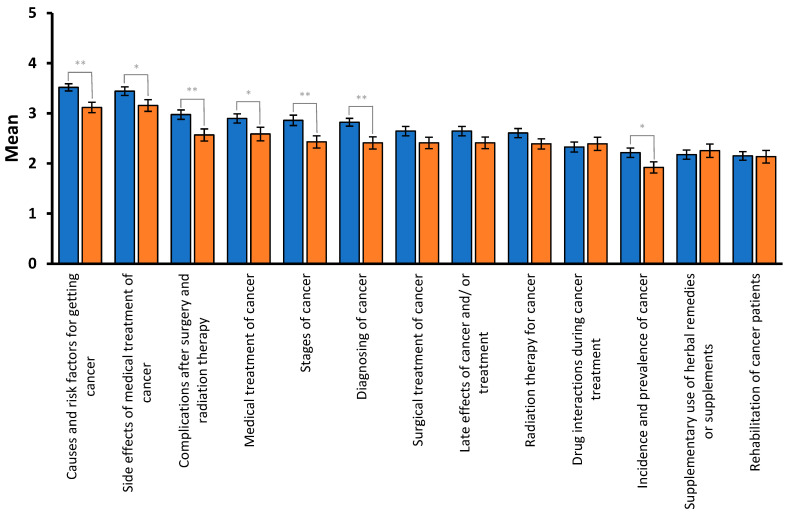
Self-perceived level of knowledge related to the topics about the cancer trajectory and cancer treatment for pharmacists (n = 79) and pharmaconomists (n = 51), respectively. Ratings for knowledge are defined as: 1 No knowledge, 2 very little knowledge, 3 some knowledge, 4 a lot of knowledge, 5 in-depth knowledge. ◼ Pharmacists. ◼ Pharmaconomists. Error bars are SEM. * *p* < 0.05, ** *p* < 0.01.

**Figure A2:**
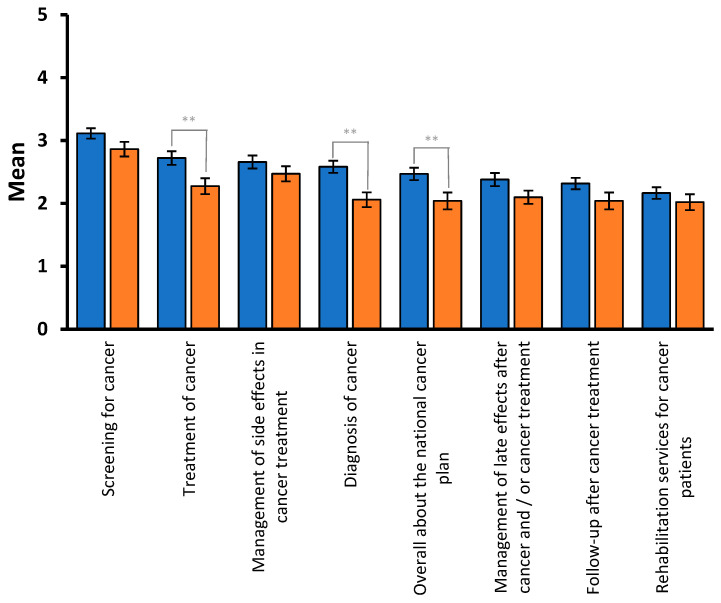
Self-perceived level of knowledge related to the topics about the organization of cancer care for pharmacists (n = 79) and pharmaconomists (n = 51), respectively. Ratings for knowledge are defined as: 1 No knowledge, 2 Very little knowledge, 3 Some knowledge, 4 A lot of knowledge, 5 In-depth knowledge. ◼ Pharmacists. ◼ Pharmaconomists. Error bars are SEM. ** *p* < 0.01.

Figure A1 depicts the respondents’ ratings of their self-perceived level of knowledge regarding 13 topics related to the cancer trajectory and cancer treatment. Figure A2 depicts the respondent’s ratings of their self-perceived level of knowledge regarding eight topics related to the organization of cancer care.

### 3.4. Barriers

**Figure 1:**
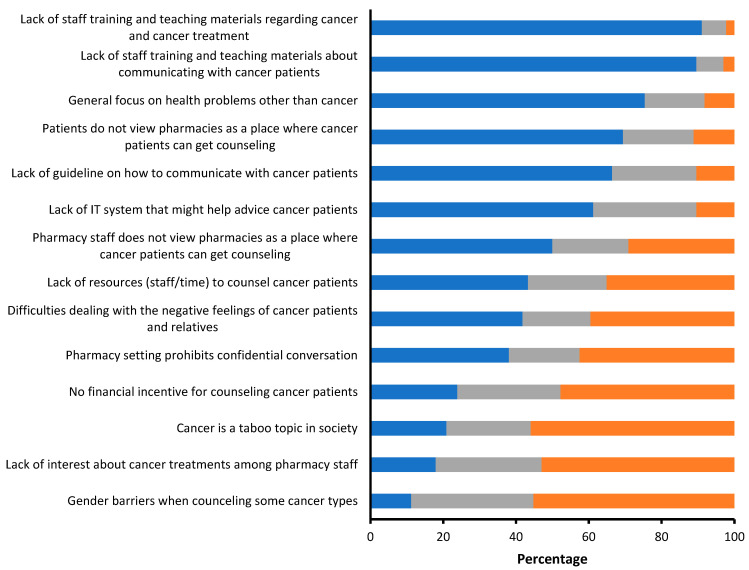
Potential barriers concerning counseling current or former cancer patients at a community pharmacy. Ratings are defined as: ◼ Agree and strongly agree. ◼ Neutral. ◼ Disagree and strongly disagree. (n = 134).

The respondents’ agreement with 14 statements referred to as barriers/challenges when counseling current or former cancer patients at the community pharmacy is presented in Figure 1.

The largest barriers reported were the lack of training and a lack of available teaching materials: 91.0% of the respondents thought that the lack of training regarding cancer and cancer treatment was a barrier preventing pharmacy staff from counseling cancer patients/survivors at a community pharmacy. Besides, 89.6% thought that the lack of training and teaching materials about communicating with cancer patients/survivors was as a barrier preventing pharmacy staff from counseling cancer patients/survivors properly. Another important barrier was the perception that pharmacy staff has more focus on other health problems than cancer at the community pharmacy (75.4%). On the other hand, more than half of the respondents did not agree that gender-related issues, cancer being a taboo subject, a lack of interest in cancer treatment among pharmacy staff, or the absence of financial initiatives to counsel cancer patients at the community pharmacy, were barriers.

### 3.5. Interest in Education

The respondents were asked whether they were interested in receiving education regarding three topics related to cancer: cancer and cancer treatment, communicating with cancer patients, and late effects of cancer. Of the 134 respondents, 91.0% were ´interested´ or ´very interested´ in learning about cancer and cancer treatment, 88.1% were ‘interested’ or ‘very interested’ in learning about how to communicate with cancer patients visiting the community pharmacy, and 93.3% were ‘interested’ or ‘very interested’ in learning about late effects of cancer.

## 4. Discussion

The study aimed at determining community pharmacy staff’s current knowledge and educational needs regarding cancer treatment modalities and organization of cancer care in Denmark and investigated whether pharmacy staff was interested in participating in education about cancer. Furthermore, the study attempted to determine barriers experienced by pharmacy staff when communicating with cancer patients/survivors at a community pharmacy. The importance of learning more about each cancer-related topic was rated between ‘important’ and ‘very important’, and the pharmacy staff was interested in participating in education about cancer. For several cancer-related topics, the pharmacists reported having more knowledge than the pharmaconomists. The largest reported barriers preventing staff from sufficiently counseling cancer patients/survivors at the community pharmacy were lack of knowledge about cancer, focus on other healthcare problems than cancer, and the perceived role of pharmacy staff.

A lack of knowledge was regarded as the largest barrier in counseling cancer patients/survivors at the community pharmacy in Denmark. Correspondingly, Hussainy et al. found that pharmacists had a low level of knowledge about cancer treatments and regarded the issue of cancer treatments, together with supplement use, as important to know more about [18]. Other studies investigating pharmacy staffs’ knowledge about skin cancer [35,36,37], prostate cancer [38], breast cancer [21,39], and bowel and breast cancer [40] also detected knowledge gaps as limiting pharmacists’ engagement in health promotion in cancer [21,35,36,37,39,40] and the counseling of cancer patients [38].

The topics with the lowest knowledge scores were the ‘rehabilitation of cancer patients’, ‘rehabilitation services for cancer patients’, and the ‘incidence and prevalence of cancer’. This might be due to the fact that these topics were beyond the scope of the pharmacists’ expertise [25]. None of these topics was regarded as the most important topics to learn about. On the other hand, this study found that information about drug interactions during cancer treatment, general knowledge about side effects, late effects, complications after treatment and how to manage them, as well as the supplementary use of herbal remedies or supplements in combination with medical cancer treatment were regarded the most important topics to learn about for pharmacy staff. It seems as if the pharmacy staff is primarily interested in medication/remedy-specific knowledge useful for everyday counseling.

When comparing pharmacists’ and pharmaconomists’ knowledge, the pharmacists had a significantly higher self-perceived level of knowledge compared to the pharmaconomists on most topics. This result is expected since pharmacy education in Denmark is a five-year university education and more theory-based compared to the pharmaconomists’ three-year education, primarily consisting of apprenticeships [5,9]. More pharmacists than pharmaconomists reported that their knowledge came from continuous education. Whereas the pharmaconomists reported obtaining more knowledge from their personal experience with cancer. However, pharmacists and pharmaconomists stated that it was important to gain more knowledge about cancer treatment in order to counsel cancer patients/survivors at the community pharmacy. Pharmacy staff’s knowledge could be improved through undergraduate oncology education and intensive continuous education programs [41,42]. Currently, oncology education for pharmacists or pharmaconomists does not exist in Denmark. Another study evaluating oncology education in undergraduate and postgraduate training programs in Canada found that oncology is underrepresented in present-day curriculums [16]. A previous study showed that the pharmaconomist program in Denmark focuses more on counseling and communication with patients than the pharmacist program [5]. Thus, it is worth investigating whether the pharmaconomists’ knowledge can be improved by providing more formal and continuous education about different chronic diseases, including cancer, in order for them to use their communication skills to the best of their ability. It is also worth discussing whether cancer care service in community pharmacies should be performed solely by pharmacists, which is the case for some of the healthcare services currently provided at community pharmacies in Denmark today [43]. A study from Ghana found that some pharmacists lack motivation, which might be caused by a lack of interest in oncology [24]. For the respondents in this study, a lack of interest in cancer treatment was not perceived as a barrier among pharmacy staff when counseling cancer patients/survivors.

This study showed that the largest barrier when counseling cancer patients/survivors was a lack of relevant training and training materials, reconfirming the results from previous studies [13,24]. Moreover, the lack of a guideline for communicating with cancer patients was also reported as a barrier. The staff at community pharmacies in Denmark uses common questioning techniques targeted toward individual patients [44]. These questioning techniques help pharmacy staff engage in dialogue with the patients, independently of the diagnosis, and ensure that they cover all relevant clinical aspects. Moreover, we believe that the communication should be patient-centered, and education on patient-centered communication should be included in both the pharmacy education program and continuous education. The implementation of this type of continuing education has recently been successfully established for community pharmacy staff in Denmark and the Netherlands [45]. Our study emphasized, however, that having a specific guideline for patient-centered communication with cancer patients might be helpful.

This study did not find that cancer is taboo for Danish pharmacy staff. However, various studies have shown that it can be a problem in society in general and during communication with healthcare professionals [46,47,48]. A reason might be that Danish pharmacy staff are used to talking about a wide variety of medications and diseases in general, which might be considered taboo subjects. Moreover, improvement in treatments and increasing cancer survival may contribute to attitudinal changes (especially among healthcare professionals), so they do not view cancer as a disease one should be reluctant to discuss. Cultural differences between countries could also be the reason for the observed differences [49]. Similarly, studies from Malaysia and Ghana found barriers due to pharmacists’ gender, where difficulties were experienced when male pharmacists would counsel breast cancer patients or if female pharmacists tried to counsel prostate cancer patients [23,24]. However, gender issues were not perceived as a barrier in the present study.

Previous studies suggested that the public might not be aware of the pharmacist’s role and that the patients still have the traditional view that a pharmacy is exclusively a place to pick up medication [21,39,40], so they do not expect to be counseled on cancer. In this study, pharmacy staff regarded this presumption as one of the largest barriers in counseling. It should be noted that this is the opinion of the pharmacy staff, so going forward, it could be worth investigating the view of the public toward community pharmacies’ role [39]. Since community pharmacists are the most easily accessible healthcare professionals, it would make sense to make the public aware of their abilities to perform functions and provide services not considered a part of their traditional roles [7,8]. Therefore, there would be a need for, e.g., campaigns to raise the awareness of the community pharmacies’ role or a cross-sectoral collaboration where the hospital will inform patients about how the community pharmacies can contribute to cancer care. Another barrier reported in this study, as well as in a previous study [13], was that community pharmacies focus on other healthcare problems rather than cancer, which might contribute to difficulty in changing public opinions about the role of pharmacies in healthcare. A reason for this focus in Denmark could be that cancer medication is dispensed at the hospital and not at community pharmacies. Experience from other countries where pharmacies contribute to cancer care provides some hope that the perception of the role of pharmacies in cancer care in Denmark can also change [3].

Last but not least, previous studies found that because community pharmacies are private businesses, they need to be profitable, so there would need to be some financial incentive for community pharmacies to provide healthcare services for cancer patients [21,22,23,24]. In this study, approximately half of the respondents did not perceive the lack of financial initiatives as a barrier. Even though a lack of financial rewards might not be a barrier, previous experiences show that when healthcare services are remunerated, pharmacies are more likely to offer the services compared to non-remunerated services [6].

### Methodological Considerations

In 2019, the total number of pharmacists and pharmaconomists working at community pharmacies in Denmark was 2698 [34], and 134 of those responded to the survey. Thus, in this study, approximately 5.0% of the community pharmacy staff completed the questionnaire. The respondents were located in all five Danish regions, and the distribution of respondents between regions corresponded to the actual distribution of pharmacies in Denmark. A limitation concerning the generalizability of the study was that the distribution of the respondents in the study did not match the distribution of staff in Danish community pharmacies. Pharmacists were better represented than pharmaconomists, and pharmacy owners were better represented than other pharmacy staff, even though the percentage of phamaconomists working in Danish community pharmacies is higher than that of pharmacists [34]. Furthermore, there are 188 pharmacy owners in Denmark, and 18 (10%) of them participated in this survey, which indicates that staff more likely to receive and respond to emails and then respond to a questionnaire were more often the pharmacy owners compared to other staff.

Another limitation was that the questionnaire used in the study had not been formally validated. However, the questionnaire included previously tested questions measuring self-assessment of the knowledge [18]. The questionnaire was also thoroughly pilot-tested in multiple rounds [28] and provided the results (i.e., pharmacists with a longer education reported more knowledge than pharmaconomists) supporting known group validity. The random order of the questions minimized question order bias [50]. Finally, the knowledge, educational needs, and barriers items in the developed questionnaire demonstrated high internal consistency reliability.

## 5. Conclusions

This study showed that community pharmacy staff in Denmark are willing to provide counseling to cancer patients/survivors, but lack relevant knowledge, and need further education, which they are interested in. The community pharmacy staff especially noted a need for more medication/remedy-specific knowledge that could be useful in their everyday work when counseling cancer patients/survivors. Consequently, the largest barrier to counseling cancer patients/survivors at the community pharmacy found in the study was a lack of knowledge and training materials. Thus, to develop a cancer counseling service for Danish community pharmacies in the future, there is a need to first of all address the identified educational gaps.

## Figures and Tables

**Table 1:** Cognitive probe questions in the interview guide [26,29].

Cognitive Probe	Question
Think-aloud	What did you think when answering this question? Was it hard/easy to understand? And why?
Comprehension	How do you understand the question?
Retrieval of information	How did you get to this answer? On what did you base your answer? Is there a specific situation you think about?
Judgment	How sure are you in answering the question?
Selection of a response to the question	How was it to answer the question? Do you think you could answer sufficiently based on how the question was posed?

**Table 2:** Characteristics of study respondents (n = 134).

Characteristics		Frequency	Percentage
Gender			
	Male	22	16.4
	Female	112	83.6
Age group (years)			
	Under 25	8	6.0
	25–34	49	36.6
	35–44	31	23.1
	45–54	17	12.7
	55–64	25	18.7
	Over 65	4	3.0
Education			
	Pharmacist	79	59.0
	Pharmaconomist	51	38.1
	Pharmacy student	4	3.0
Job title			
	Pharmacy owner	18	13.4
	Pharmacy manager	22	16.4
	Information-, quality-, prescription pharmacist	29	21.6
	Practicing pharmacist	19	14.2
	Practicing pharmaconomist	42	31.3
	Pharmacy student under an internship	4	3.0
Region			
	North Denmark region	12	9.0
	Central Denmark Region	26	19.4
	Region of Southern Denmark	28	20.9
	Region Zealand	21	15.7
	Capital Region of Denmark	47	35.1
Years of Practice			
	Less than 1 year	4	3.0
	1–3 years	29	21.6
	4–10 years	33	24.6
	11–20 years	33	24.6
	More than 20 years	35	26.1

**Table 3:** Indications of where respondent’s knowledge about cancer came from.

Knowledge of Cancer from	Pharmacists (n = 79)		Pharmaconomists (n = 51)	
	Frequency	Percentage	Frequency	Percentage
No knowledge	3	3.8	4	7.8
Pharmacist-/pharmaconomist education	50	63.3	25	49.0
Continuous education (e.g., courses)	14	17.7	3	5.9
Work experience	37	46.8	23	45.1
Self-study	29	36.7	18	35.3
Cancer trajectories I have experienced or been close to (e.g., own or relatives’)	42	53.2	33	64.7
Other	7	8.9	4	7.8

**Table 4:** Self-perceived level of knowledge and importance of learning more about topics related to the cancer trajectory and cancer treatment, and the organization of cancer care (n = 134).

Category ^a^	Topic	Knowledge			Importance		
		Mean ^b^	CI (95%)	SD	Mean ^c^	CI (95%)	SD
Treatment	Causes and risk factors for getting cancer	3.4	[3.24; 3.49]	**	3.4	[3.28; 3.60]	
-	Stages of cancer	2.7	[2.55; 2.87]	**	3.1	[2.89; 3.21]	
-	Incidence and prevalence of cancer	2.1	[1.97; 2.24]	*	2.6	[2.48; 2.79]	
-	Diagnosing of cancer	2.7	[2.53; 2.80]	**	3.2	[2.74; 3.05]	
-	Surgical treatment of cancer	2.6	[2.42; 2.70]		2.7	[2.56; 2.90]	
-	Radiation therapy for cancer	2.5	[2.38; 2.65]		2.9	[2.75; 3.09]	
-	Medical treatment of cancer	2.8	[2.65; 2.96]	*	3.8	[3.59; 3.91]	
-	Side effects of medical treatment of cancer	3.3	[3.21; 3.48]	*	4.3	[4.13; 4.40]	
-	Complications after surgery and radiation therapy	2.8	[2.68; 2.98]	**	3.8	[3.68; 3.99]	
-	Drug interactions during cancer treatment	2.4	[2.20; 2.51]		4.4	[4.28; 4.53]	
-	Supplementary use of herbal remedies or supplements at the same time as medical cancer treatment	2.2	[2.08; 2.37]		4.1	[3.99; 4.28]	
-	Rehabilitation of cancer patients	2.2	[2.03; 2.30]		3.0	[2.84; 3.16]	
-	Late effects of cancer and/ or treatment	2.6	[2.44; 2.73]		3.7	[3.54; 3.83]	
Organization	Overall about the national cancer plan	2.3	[2.15; 2.47]	**	3.0	[2.88; 3.17]	
-	Screening for cancer	3.0	[2.89; 3.16]		3.0	[2.83; 3.14]	
-	Diagnosis of cancer	2.4	[2.25; 2.55]	**	2.9	[2.74; 3.05]	
-	Treatment of cancer	2.5	[2.38; 2.71]	**	3.2	[3.04; 3.37]	
-	Rehabilitation services for cancer patients	2.1	[1.96; 2.25]		3.2	[2.99; 3.31]	
-	Follow-up after cancer treatment	2.2	[2.06; 2.36]		3.1	[2.96; 3.28]	
-	Management of side effects in cancer treatment	2.6	[2.44; 2.75]		4.1	[4.00; 4.27]	
-	Management of late effects after cancer and/or cancer treatment	2.3	[2.14; 2.44]		3.9	[3.76; 4.03]	

^a^ The topics are divided into categories/sections about either the cancer trajectory and cancer treatment or the organization of cancer care. ^b^ Ratings for knowledge are defined as: 1 No knowledge, 2 very little knowledge, 3 some knowledge, 4 a lot of knowledge, 5 in-depth knowledge. ^c^ Ratings for importance are defined as: 1 Not important, 2 less important, 3 important, 4 very important, 5 extremely important. * *p* < 0.05, ** *p* < 0.01 for the SD (Significant difference) between pharmacists (n = 79) and pharmaconomists (n = 51).

## Data Availability

The data presented in this study can be obtained upon request from the corresponding author.
